# Noncosmetic Periocular Therapeutic Applications of Botulinum Toxin

**DOI:** 10.4103/0974-9233.63069

**Published:** 2010

**Authors:** Pelin Kaynak-Hekimhan

**Affiliations:** Istanbul Beyoğlu Eye Research and Training Hospital, Galata, Istanbul, Turkey

**Keywords:** Apraxia of Eyelid Opening, Blepharospasm, Botulinum Toxin, Eyelid Retraction, Periocular Applications, Synkinetic Eyelid Movements

## Abstract

Botulinum toxin blocks acetylcholine release at the neuromuscular junction. The drug which was initially found to be useful in the treatment of strabismus has been extremely effective in the treatment of variety of conditions, both cosmetic and noncosmetic. Some of the noncosmetic uses of botulinum toxin applications include treatment of spastic facial dystonias, temporary treatment of idiopathic or thyroid dysfunction-induced upper eyelid retraction, suppression of undesired hyperlacrimation, induction of temporary ptosis by chemodenervation in facial paralysis, and correction of lower eyelid spastic entropion. Additional periocular uses include control of synchronic eyelid and extraocular muscle movements after aberrant regeneration of cranial nerve palsies. Cosmetic effects of botulinum toxin were discovered accidentally during treatments of facial dystonias. Some of the emerging nonperiocular application for the drug includes treatment of hyperhidrosis, migraine, tension-type headaches, and paralytic spasticity. Some of the undesired side effects of periocular applications of botulinum toxin inlcude ecchymosis, rash, hematoma, headache, flu-like symptoms, nausea, dizziness, loss of facial expression, lower eyelid laxity, dermatochalasis, ectropion, epiphora, eyebrow and eyelid ptosis, lagophthalmos, keratitis sicca, and diplopia.

## INTRODUCTION AND HISTORY

Botulinum toxin is a powerful toxin, which prevents muscular contraction by blocking acetylcholine release at the neuromuscular junction. This toxin, which is used predominantly in treatments of muscle dystonias with spasms and for cosmetic reasons, was first used by Scott *et al*. in 1973 in strabismus patients.^1^ Food and Drug Administration (FDA) approved botulinum toxin use for strabismus patients in 1979. FDA approval for the treatment of hemifacial spasm and blepharospasm was in 1989 although botulinum toxin was utilized for blepharospasm treatment since 1982. Cosmetic effects of botulinum toxin were discovered accidentally during treatments of facial dystonias.^2^ Carruthers and Carruthers published several studies related to the cosmetic applications of botulinum toxin, proving that it decreased the depth and appearance of the kinetic facial lines.^3^–^5^ In 2002, the FDA approved the use of botulinum toxin A for the treatment of glabellar lines. Botulinum toxin is also used extensively for crowsfeet, and in the perioral area, nose, chin, and neck areas.^6^ Intramuscular botulinum toxin injections, frequently applied in treating spastic facial dystonias are currently the most preferable methods of treatment due to undesired effects of alternative treatment methods.^7^–^14^ Botulinum toxin is effective in the temporary treatment of idiopathic and thyroid dysfunction-induced upper eyelid retraction.^15^–^18^ Botulinum toxin injections in the lacrimal gland have been found to be effective in the treatment of hyperlacrimation due to various causes. Additionally the temporary induction of ptosis to provide corneal protection in facial paralysis has been found to be very beneficial. Some of the other areas of use for this toxin include hyperhidrosis, migraine, tension-type headaches, and paralytic spasticity.^10^

### Botulinum toxin

Botulinum toxin is the poisonous exotoxin of *Clostridium*. The bacterium *Clostridium botulinum* produces eight antigenically distinct exotoxins. Serologic types include A, B, C, D, E, F, and G. Type E is also produced by *C. butyricum*. Type F is produced by *Clostridium baratii*.^19^ Type A, B, and E botulinum toxins are colorless, odorless, and tasteless. Only these three types of toxins affect humans and can cause systemic botulismus. Type A is the most potent toxin, followed by types B and F. Each botulinum toxin is synthesized as a single-chain protein, which is inactive until it is cleaved by bacterial proteases into its active form. The active botulinum toxins are composed of two chains: one heavy chain joined to a light chain by a relatively weak disulfide bond, which is shown to be highly responsible for the instability of the molecule. The toxin is inactivated by heat, 85°C (185°F) or greater in 5 min.^19^–^23^

### Mechanism of action

Botulinum toxin blocks the release of acetylcholine from vesicles at the presynaptic nerve terminal. It also inhibits release of acetylcholine at the autonomic ganglia, postganglionic parasympathetic, and sympathetic nerve endings. The different serotypes bind to different sites on the motor neuron terminal and within the motor neuron. The heavy chain functions both as a channel and a companion to bring the light chain across the endosomal membrane and then into the cytosol in the presynaptic region. The light chain acts inside the cell on synaptosomal-associated protein receptor proteins (SNARE) to block the release of the vesicle-bound neurotransmitter acetylcholine from nicotinic and muscarinic nerve endings. Muscle weakness becomes evident in 2–4 days due to the continued release of acetylcholine from vesicles that have not been blocked by the toxin. Recovery of muscle activity typically begins 3–4 months after injection and is thought to occur due to the regeneration of new endplate units.^23^

### Commercial preparations

Doses of all commercially available forms of botulinum toxin are expressed in terms of units (mouse units). The standard measurement of the potency of the toxin is one international unit (IU), which is the amount of toxin that kills 50% of a group of 18–20 female Swiss-Webster mice (LD50) when injected intraperitonally. The LD50 in humans is estimated to be approximately 2730 IU^19^–^21^ BOTOX^®^ (Allergan Corporation, Irvine, CA, USA) is a dry, protein crystalline complex of botulinum toxin A which contains 100 units per bottle. One unit of BOTOX^®^ COSMETIC equals to the calculated median intraperitoneal lethal dose (LD50) in mice.^19^ The product is unstable so it must be kept frozen before constitution. The recommended doses are usually prepared by diluting the contents of bottle with 2 ml of sterile saline without preservative, leaving 25 U for each 0.5 ml of solution. One nanogram contains 2.5 IU.^22^ Dysport (Ipsen, Slough, UK) is another trademark of botulinum toxin type A.^20^

The onset of paralysis takes 24–48 h and maximum paralysis is acheived at 7–10 days. The effect usually lasts 4–6 months. Repeated injections may delay the onset of paralysis, but sometimes a more protracted paralysis will occur.^20^ Approximately, 4 U of Dysport are equivalent to 1 U of BOTOX^®^.^22^

Myobloc^®^ (Neurobloc; Elan Pharmaceuticals, San Diego, CA, USA) contains a liquid formulation of purified botulinum toxin type B. When reconstituted, Myobloc^®^ has a shelf life of more than 12 months which is longer than BOTOX^®^. It has a faster onset of action and better diffusion into tissues; however, the injections are more painful due to the acidity of the product. Overall, botulinum toxin A is 50–100 times more potent than botulinum toxin B.^24^

### Reconstitution and storage

Botulinum toxin A is recommended to be reconstituted with sterile nonpreserved 0.9% NaCl solution before injection and must be kept at 4°C until injection. It has to be injected within 4 h after reconstitution for maximum activity. The weak disulfide bonds between the two chains of the toxin render it fragile under mechanical stress such as frothing when diluting and agitating the liquid inside the vial. Table 1 lists the most common used botulinum toxin preparations and their storage conditions.

**Table 1:** Botulinum Toxin. Storage and preparation conditions

	Storage temperatures		Duration of activity
		
	Before reconstitution	After reconstitution	
Botulinum toxin A			
Botox^®^:	−5°C	2-8 °C	4 hours
Botox cosmetic^®^:	−5°C	2-8 °C	4 hours
Dysport^®^:	2-8 °C	2-8 °C	1 hour
Botulinum toxin B			
Neuroblock^®^:	2-8 °C	Room temperature	8 hours
Myoblock^®^:	Room temperature	Room temperature	Up to 30 months

The concentration of the botulinum toxin depends on the amount of diluent in the vial which can be determined by the physician. In clinical applications preferred dilution is generally 50 U/ml when used for the management of blepharospasm and hemifacial spasm and 100 U/1 ml for cosmetic applications around the eyes. Table 2 shows the toxin concentration in 0.1 ml of two commonly used commercial forms of botulinum toxin A with various volumes of diluent. Tuberculin syringes with 30-gauge needles are preferred which allow more painless and accurate injections at intended sites with relatively low risk of bleeding. When botulinum toxin is injected in the periocular area, both ice packs and EMLA^®^ cream can be applied for topical anesthesia. Elibol *et al*. reported that EMLA^®^ cream for periocular anesthesia may work slightly better than ice packs for skin cooling when botulinum toxin A is injected.^25^

**Table 2:** Botulinum toxin A. Concentration with various volumes of diluent used

0.9% NaCl added (ml)	Botox^®^ dose (U/0.1ml)	Dysport^®^ dose (U/0.1ml)
1	10	50
2	5	25
4	2.5	12.5
8	1.25	6.25
10	1	5

### Therapeutic uses of botulinum toxin in periocular area

Facial muscle dyskinesias such as benign essential blepharospasms, hemifacial spasms, Meige syndrome, apraxia of eyelid opening and orbicularis myokimia, and synkinetic eyelid movements are manageable with botulinum toxin injections. CNS depressants,^26^ orbicularis myectomy, and selective facial nerve neurectomy are alternative treatments. Botulinum toxin injections in orbicularis oculi, corrugator superciliaris, and occasionally into frontalis muscles have been found to be very effective for the treatment of blepharospasm.^6^–^13^ Muscles involved in facial dyskinesias must be correctly evaluated on physical examination. Any accompanying pathologies have to be investigated and consultations with other specialists sought prior to any treatment recommendations. Electromyographic examination may be helpful in localizing the involuntarily contracting muscles in cases when the muscle to be injected is not localized accurately. Video documentations before and after the botulinum toxin treatment may be more useful than documentary photographs.

### Botulinum toxin in blepharospasm and hemi facial spasm

Benign essential blepharospasm is the involuntary, repetitive contractions of orbicularis oculi muscle. Depressor muscles of eyebrows such as corrugator superciliaris and procerus are also involved. Reflex blepharospasm due to dry eye must not be mistakenly diagnosed as benign essential blepharospasm. Meige syndrome is a cranial dystonia with bilateral blepharospasm accompanied by dystonia in the lower face. Involuntary contractions of orbicularis oris, buccinator, and masseter muscles are prominent. Hemifacial spasm is characterized by unilateral repetitive contractions of facial muscles. It may result from compression of fibers of seventh nerve root. Posterior fossa tumors may be the underlying cause of this condition. Orbicularis myokimia denotes a condition in young individuals with involuntary twitches of some orbicularis oculi muscle fiber bundles. Chronic stress, fatigue, and caffeine and alcohol intake may exacerbate the frequency and severity of spasms.

**Figure 1:**
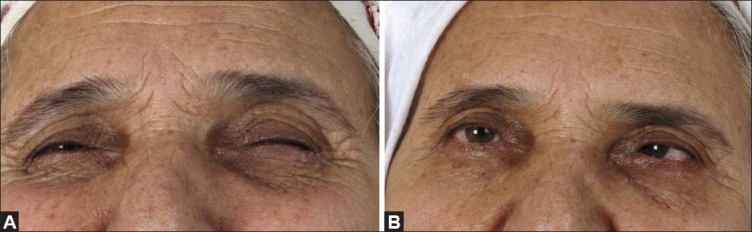
Photographs of a patient with blepharospasm before (A) and after (B) botulinum toxin A injection

The usual dose and muscles injected for blepharospasm treatment is about 2–5 U in each of the three points laterally in orbicularis oculi muscle 1 cm lateral to the lateral orbital rim. Subcutaneous injections of 2 U botulinum toxin A into medial to upper and lower eyelids in the orbicularis oculi preseptal fibers, but not into the pretarsal fibers in this area may be delivered. The injection sites must be at least 5 mm away from the lacrimal punctae to avoid lacrimal pump failure. Botulinum toxin A injections of 1–3 units into frontalis muscles centrally and 5–10 U in the corrugator supercilii muscles and 1–2 U in procerus muscles administered, generally may stop contractions for 4–6 months [Figure 1]. Patients are seen in the second week post-injection for evaluation of the efficacy, side effects, and secondary effect of the treatment. The need for repeat injections may be evaluated at this visit. Doses and needs for modifications for the future injections assessed during this visit.

Patients with hemifacial spasms may need botulinum toxin injections in lower orbicularis muscles, and occasionally in the lower facial muscles. Doses may vary individually, being lower than those for blepharospasm. Meige syndrome is treated with higher doses. Orbicularis myokimia treatment requires lower dose injections of the toxin. Response to the treatments with botulinum toxin continues after repeated injections in the majority of cases even when followed up for more than 10 years.^8^ For patients who are nonresponsive to botulinum toxin A may benefit from botulinum toxin B can be beneficial in treating the spasms.^9^ Alternately, injecting higher doses of the toxin may also stop involuntary contractions that do not respond to lower doses.^27^

Temporary eyelid and facial ptosis, lagophthalmos, and epiphora are the undesired effects of the treatment. Diplopia may occur as a result of diffusion of toxin into extraocular muscles.

### Botulinum toxin in apraxia of eyelid opening

Apraxia of eyelid opening is the inability to initiate opening of the eyelids due to difficulty in overcoming levator palpebrae inhibition. It may be noted in patients with blepharospasm due to pretarsal fibers of orbicularis oculi muscle activity. Injections of 0.5–2 U of botulinum toxin A on two sides medially and laterally 5 mm away from the lid margin may be effective in decreasing apraxia of eyelid opening. If the frontalis muscle is functioning, these patients may benefit from frontalis suspension surgery.

### Botulinum toxin for dysthroid and upper eyelid retraction management

**Figure 2:**
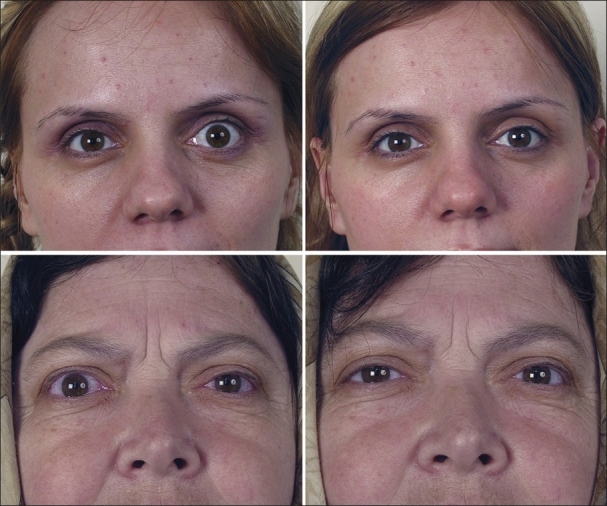
Photographs of two patients with upper eyelid retraction before (left) and after (right) botulinum toxin A injection

**Figure 3:**
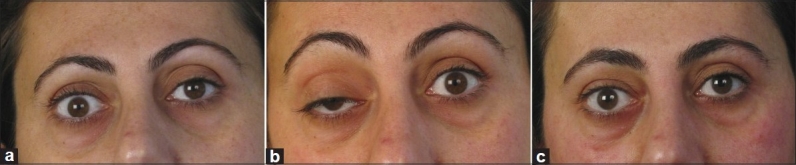
Patient with lagophthalmus (a) had ptosis complication (b) after botulinum toxin injections and returned to normal after 4 weeks with the correction of lagophtalmos (c)

In 1996, Özkan *et al.* investigated the effectiveness of botulinum toxin A in the treatment of dysthyroid upper eyelid retraction and reported its efficacy in the lowering of retracted eyelids.^15^ Botulinum toxin administered for temporary correction, particularly in ascending stage of Rundle's curve and during the period when stabilization is expected, and radical surgical management is delayed for more accurate outcome. [Figure 2] Generally, 1–10 U of botulinum toxin A is injected subconjunctivally at the upper border of tarsus. The dose may be divided into two injections, medially and laterally to minimize exaggerated eyelid ptosis as an undesired side effect of the toxin^28^,^29^ Despite multiple administrations of botulinum toxin A, the effect is temporary. If the upper eyelid retraction persists despite patient's becoming euthyroid, levator recession surgery with or without spacer materials may be required. Morgensten *et al.* achieved decrease in eyelid aperture in 94% of the cases by administering 2.5–10 U botulinum toxin A injections transconjunctivally in one or two points of levator–Müller's muscle complex from the upper side of the tarsus.^18^ Shih *et al*. have also achieved similar results by injecting botulinum toxin A through skin into levator muscle.^30^ Upper eyelid ptosis is the most frequently seen undesired effect of such injections, and it is temporary [Figure 3].

**Figure 4:**
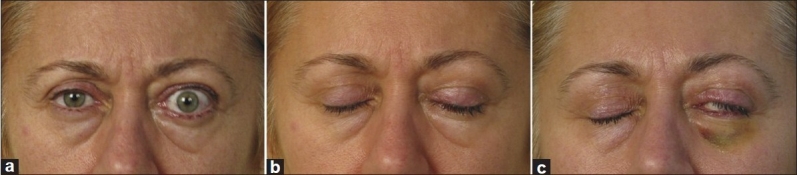
Patient with lagophthalmus (a) who had good closure of upper eyelid (b) had insufficient eyelid closure complication (c) after botulinum toxin injections, due to diffusion of the toxin into orbicularis oculi muscle

Lagophthalmus becomes more evident if botulinum toxin diffuses into the orbicularis oculi muscle fibers [Figure 4]. Corneal protective measures are mandatory in case of this complication. Olver has also reported successful results in decreasing the activity of corrugator supercilia muscles in cases with dysthyroid ophthalmopathy by injecting botulinum toxin into these muscle groups.^16^

### Botulinum toxin for entropion treatment

Botulinum toxin injection decreases the tone of lower pretarsal and preseptal fibers of orbicularis oculi overriding and therefore correcting spastic entropion temporarily. Roughly, 1–5 U of botulinum toxin injections to the central portion of the subciliary orbicularis muscle 3–5 mm inferior to the eyelid margin in lower eyelid treats the spastic component of entropion lasting for 4–5 months. Injections of botulinum toxin in infants are not commonly performed.

Christiansen *et al.* injected botulinum toxin A on the lower eyelid of a 3-week-old child with congenital entropion which was the cause of corneal ulcers of the infant.^31^ There were no undesired side effects from botulinum toxin A, and the child's ulcer healed without the need for any surgery. Gustatory lacrimal gland function (crocodile tear syndrome) can be controlled by two.5–20 U of botulinum toxin A injection administered in the lacrimal gland; however, side effects such as eyelid ptosis and dry eye symptoms can occur.^31^–^34^

### Botulinum toxin for corneal protection in facial paralysis

For cases with facial paralysis, particularly for patients for whom a surgical procedure seems to be difficult, lagophtalmos can be decreased by achieving eyelid ptosis with 2–10 U of botulinum toxin A injection in levator palpebrae superioris muscle. Such treatment may be desired instead of tarsorrhaphy and/or gold weight implantation for the treatment of corneal ulcers due to exposure keratopathy.^35^ Patients who receive radiation therapy near the face are also good candidates for similar application of botulinum toxin because the atrophied eyelid skin would not tolerate an eyelid implant over the long term. Usually 5–15 U of botulinum toxin A is injected in the levator muscle subconjunctivally 5–6 mm above the tarsus to prevent diffusion into orbicularis oculi muscle fibers and worsen the lagophthalmos in these patients.

### Control of synkinetic eyelid movements

Synchronic movements of eyelid retractor and protractors as well as extraocular muscles can be seen after aberrant regeneration following third or seventh cranial nerve palsies. The muscle contraction can be controlled by customized doses of botulinum toxin injections in these muscles. Chua *et al.* administrated 40–120 U botulinum toxin A (Dysport) injections on orbicularis oculi muscle of five patients in order to limit the synkinetic eyelid movements occurring after aberrant seventh nerve paralysis.^36^ They was observed that synkinetic movements decreased for 3 months both objectively and subjectively in all cases. Ptosis observed in two patients, but this side effect was not seen in patients treated with lower doses.^36^

### Complications of botulinum toxin in periocular procedures

General complications include ecchymosis, rash, hematoma, headache, flu-like symptoms, nausea, and dizziness. Most common ocular complications are undercorrection, asymmetrical features, change in and/or loss of facial expression (overcorrection), lower eyelid laxity, dermatochalasis, ectropion, epiphora, eyebrow and eyelid ptosis, lagophthalmos in closing due to orbicularis muscle weakness, keratitis sicca, diplopia, photophobia, decrease in visual acuity, and higher intraocular pressure.^37^

Perilabial weakness related to weakening of the zygomaticus major muscle can occur when botulinum toxin is injected for crow's feet treatment.^38^ Festoon formation in recovered cases with blepharoplasty is another reported botulinum toxin complication. Some of the reasons may be decreased lymphatic drainage due to the decreased tone of orbicularis muscles of the patients involved, loss of “pumping action” of the muscle, and fluid accumulation in loose soft tissue over the zygoma.^39^

Another complication that could be caused by anticholinergic effects of botulinum toxin is high-intraocular pressure due to possible angle closure occurring as a result of anticholinergic effects when reaching the ciliary ganglion.^37^

Eyebrow ptosis and dermatochalasis are frequently seen complications of periocular injections of botulinum toxin. Careful patient selection, low volume/high concentration applications, and adding adrenaline in the injection may decrease frequency of complications.^40^

Orbicularis oculi muscle lies subdermally in a circular fashion surrounding the palpebral aperture, and its main function is closing the eye. This muscle also contributes highly to the lacrimal pump mechanism in drainage of the tears down the nasolacrimal duct to the nasal cavity. Temporal injections subcutaneously target the orbicularis oculi fibers, especially causing the crowsfeet rhytids which are one of the first signs of aging. Injections in the upper temporal portion of the orbital fibers of the orbicularis oculi muscle lift the lateral end of the eye brow and decrease the dermatochalasis in this area.^40^–^43^

Injecting botulinum toxin A in the subciliary pretarsal fibers of orbicularis oculi muscles must be done cautiously because paralysis of the tarsal orbicularis associated with higher doses may lead to scleral show and occasional epiphora. Injections of 1–5 U botulinum toxin A at each point, at least 1 cm lateral to the orbital rim may avoid the diffusion of the toxin to the extraocular muscles, thus decreasing complications such as diplopia. Injecting too close to the lid margins may lead to insufficent eyelid closure, reflex tearing, and sometimes corneal erosions. Botulinum toxin leaking into extraocular muscles might induce diplopia,^44^ and it might also cause decreased lacrimal excretion, corneal ulcers and reduced visual acuity due to high doses of botulinum toxin diffusing into the posterior septum and infiltrating the lacrimal gland.^45^ Another paradoxical recommendation for botulinum toxin is its induction of pretarsal orbicular muscle weakness to decrease lacrimal drainage effect, which might help dry eye. However, in the predisposed patients, lots of the eyelid corneal interface could worsen the dry eye symptoms.^46^

### Contrindications

Pregnancy and lactation, neuromuscular junction disorders (*Myastenia gravis*), peripheral motor neuropathies, active infections, hypersensitivity to any of the contents are the contraindications of botulinum toxin.

### Botulinum toxin, botulismus, antibody development, and non responders

As the doses are low and intervals are relatively long, there is only one reported case of botulism development after botulinum toxin A injection in a 47-year-old female patient for cosmetic purposes.^47^ Although her recovery period was prolonged, there was complete recovery from this very rare and unusual side effect of botulinum toxin A. One of the most important issues that arises in relation to the increasing number of cosmetic procedures is antibody development against botulinum toxin and the concern for not receiving proper response after the treatment.^48^ For cases refractory to botulinum toxin A injections, other botulinum toxin subtypes might offer some hope.^49^–^51^ However, pharmacological effects and duration of activity of such various subtypes are different from botulinum toxin A. Currently, Botox and Dysport are considered the most effective and reliable preparations.^52^

Cross tolerance may occur between various subtypes of the toxin. Berwick *et al*. reported a case of an 8-year-old child who did not respond to the botulinum toxin B injections after three attempts, also became refractory to botulinum toxin A preparations.^53^

A meta-analysis study, Dutton reported that 6% of cases did not respond to their first injection of botulinum toxin A.^54^ Unlike these cases, patients who responded when the dose was increased or who did not respond despite response to previous treatments developed secondary antibodies and neutralized botulinum toxin molecules. This phenomenon should not be confused with nonresponders, and these patients should be treated with different botulinum toxin subtypes.^55^

### Debatable issues about dilution and storage

According to the manufacturer's recommendation, botulinum toxin significantly loses its activity within a finite period after reconstitution. The molecule separates into pieces due to fracture of the protein chains. The protein structure is connected with weakened disulfide bonds that can break as a result of heat and/or agitation. Therefore the toxin should be transported using cold chain standards without vibration. However, Trinidad *et al*. reported in their controlled double blinded study that the molecular structure of botulinum toxin A was resistant to foaming and the cosmetic effect lasted for the same period.^56^ Studies by various authors have demonstrated that reconstitution of botulinum toxin 1 or 2 weeks prior to injections did not decrease the efficacy.^57^–^59^ Similarly, Hexsel *et al*. reported that botulinum toxin molecule reserved its effectiveness upto 6 weeks after reconstitution. Additional pharmacologic studies maybe necessary to address this controversy.^60^

The botulinum toxin manufacturers recommend reconstitution of botulinum toxin type A using nonpreserved saline. However, less painful injections have been noted with the use of the preserved saline compared to nonpreserved preparation. The preserved reconstitution appeared to have no effect on clinical outcomes.^61^ Studies by van Laborde *et al*.^62^ and Alam *et al*.^63^ also prove that use of preservative-containing saline to further dilute botulinum toxin types B and A, respectively, can significantly decrease patient discomfort during injection. The effect does not change with the preservative content of the diluent. Short- and long-term results show equal effectiveness of botulinum toxin A, whether reconstituted in saline or in lidocaine. Injections of botulinum toxin A reconstituted in lidocaine are associated with significantly reduced pain and lidocaine-reconstituted botulinum toxin A may be preferable for treating axillary hyperhidrosis.^64^

## CONCLUSION

The therapeutic use of botulinum toxin for the pathologies in the periocular region is repeatable, safe, and temporarily effective. Potential complications should be discussed with the patient before injection procedures and the patients should be selected carefully. Determining optimum doses for certain anatomic areas, ideal concentrations, dose responses among different botulinum toxin serotypes, prevention of antigen formation, possible efficacy changes in long-term treatments, and long-term reliability are the areas that may require additional research.
